# The risk of angiosarcoma following primary breast cancer

**DOI:** 10.1038/sj.bjc.6690726

**Published:** 1999-10

**Authors:** W Cozen, L Bernstein, F Wang, M F Press, T M Mack

**Affiliations:** University of Southern California School of Medicine, Department of Preventive Medicine, USC/NOR MS 44, 1441 Eastlake Avenue, Los Angeles, CA, 90033, USA; Department of Pathology, University of Southern California School of Medicine, Los Angeles, California

**Keywords:** breast cancer, Stewart–Treves syndrome, lymphangiosarcoma, haemangiosarcoma, angiosarcoma, radiation, lymphoedema

## Abstract

Lymphangiosarcoma of the upper extremity is a rare and aggressive tumour reported to occur following post-mastectomy lymphoedema (Stewart–Treves syndrome). Haemangiosarcoma, a related rare tumour, has occasionally been reported to occur in the breast following irradiation. We conducted a case-control study using the University of Southern California-Cancer Surveillance Program, the population-based cancer registry for Los Angeles County, to evaluate the relationship between invasive female breast cancer and subsequent upper extremity or chest lymphangiosarcoma and haemangiosarcoma together referred to as angiosarcoma. Cases were females diagnosed between 1972 and 1995 with angiosarcoma of the upper extremity (*n* = 20) or chest (*n* = 48) who were 25 years of age or older and residing in Los Angeles County when diagnosed. Other sarcomas at the same anatomic sites were also studied. Controls were females diagnosed with cancers other than sarcoma during the same time period (*n* = 266 444). Cases and controls were then compared with respect to history of a prior invasive epithelial breast cancer. A history of breast cancer increased the risk of upper extremity angiosarcoma by more than 59-fold (odds ratio [OR] = 59.3, 95% confidence interval [95% CI] = 21.9–152.8). A strong increase in risk after breast cancer was also observed for angiosarcoma of the chest and breast (OR = 11.6, 95% CI = 4.3–26.1) and for other sarcomas of the chest and breast (OR = 3.3, 95% CI = 1.1–1.7). © 1999 Cancer Research Campaign


*British Journal of Cancer* (1999) **81**(3), 532–536
© 1999 Cancer Research Campaign
Article no. bjoc.1998.0726

### The risk of angiosarcoma following primary breast cancer

**W Cozen[1], L Bernstein[1], F Wang[1], MF Press[2] and TM Mack[1]**

[1]University of Southern California School of Medicine, Department of Preventive Medicine, USC/NOR MS 44, 1441 Eastlake Avenue, Los Angeles, CA 90033, USA; [2]University of Southern California School of Medicine, Department of Pathology, Los Angeles, California.

**Summary** Lymphangiosarcoma of the upper extremity is a rare and aggressive tumour reported to occur following post-mastectomy lymphoedema (Stewart–Treves syndrome). Haemangiosarcoma, a related rare tumour, has occasionally been reported to occur in the breast following irradiation. We conducted a case-control study using the University of Southern California-Cancer Surveillance Program, the population-based cancer registry for Los Angeles County, to evaluate the relationship between invasive female breast cancer and subsequent upper extremity or chest lymphangiosarcoma and haemangiosarcoma together referred to as angiosarcoma. Cases were females diagnosed between 1972 and 1995 with angiosarcoma of the upper extremity ($n$ = 20) or chest ($n$ = 48) who were 25 years of age or older and residing in Los Angeles County when diagnosed. Other sarcomas at the same anatomic sites were also studied. Controls were females diagnosed with cancers other than sarcoma during the same time period ($n$ = 266 444). Cases and controls were then compared with respect to history of a prior invasive epithelial breast cancer. A history of breast cancer increased the risk of upper extremity angiosarcoma by more than 59-fold (odds ratio [OR] = 59.3, 95% confidence interval [95% CI] = 21.9–152.8). A strong increase in risk after breast cancer was also observed for angiosarcoma of the chest and breast (OR = 11.6, 95% CI = 4.3–26.1) and for other sarcomas of the chest and breast (OR = 3.3, 95% CI = 1.1–1.7). © 1999 Cancer Research Campaign

**Keywords:** breast cancer; Stewart–Treves syndrome; lymphangiosarcoma; haemangiosarcoma; angiosarcoma; radiation; lymphoedema

Angiosarcoma is a generic term that includes malignant sarcomas originating from either lymphatic or capillary endothelium, namely lymphangiosarcoma and haemangiosarcoma respectively. Reports of lymphangiosarcoma in association with lymphoedema of an extremity can be found as early as 1906 (Lowenstein, 1906). The occurrence of lymphangiosarcoma in a setting of post-mastectomy upper extremity oedema was originally described in a report of six cases in 1948 (Stewart and Treves, 1948), and has since been designated the Stewart–Treves syndrome. By 1970, 162 cases of this syndrome had been reported worldwide in case reports and hospital case series (Sanchez et al, 1989).

Haemangiosarcoma of the chest or breast has been described in at least two different clinical settings: in very young women with no prior cancer (McClanahan and Hogg, 1954; Steingaszner et al, 1965) and in older women following irradiation of the breast for epithelial breast cancer (Kuten et al, 1985; Rubin et al, 1990; Mark et al, 1994).

Lymphangiosarcoma and haemangiosarcoma are often described together in older reports because of the high degree of misclassification (Woodward et al, 1972; Maddox and Evans, 1981; d'Amore et al, 1990; Karpeh et al, 1991). In spite of recently developed immunohistochemical antibodies, the distinction remains difficult and even arbitrary, and the two tumours are often categorized together as angiosarcoma. In fact, immunohistochemical and electron microscopic studies offer equal evidence for haemangiomatous origin of lymphangiosarcoma (Enzinger and Weiss, 1995). The average annual age-adjusted incidence rate of angiosarcoma for white females in the USA is 1.6/100 000 and tumours of the upper extremity and breast account for 25% and 44% respectively, of these sarcomas (Mack, 1995).

No formal attempt has been made previously to quantify the relationship between invasive breast cancer and subsequent risk of angiosarcoma, possibly because of the rarity of the tumours. We conducted a case-control study within a population-based cancer registry to evaluate this relationship.

*Received 20 August 1998*
*Revised 4 January 1999*
*Accepted 28 January 1999*

*Correspondence to:* W Cozen

### MATERIALS AND METHODS

The University of Southern California Cancer Surveillance Program (USC-CSP), the population-based cancer registry for Los Angeles County (LAC), has been actively collecting information on newly diagnosed invasive cancer cases since 1972 (Mack, 1977). For a given patient, each new primary cancer diagnosis is reported separately; recurrences are not considered new diagnoses and are not reported. The majority of diagnoses are based on pathology reports (over 96%), with the remainder based on death certificates or clinical diagnoses. Pathological diagnoses are not validated by a second histological review; with over 1 million cases of cancers diagnosed in Los Angeles since 1972, the task would be unrealistic.

The USC-CSP registry emphasizes collection of descriptive incidence data based on variables such as race, age, gender, birthplace, religion, ethnicity and social class. Collection of survival data began in 1992, when the registry became a Surveillance,

Epidemiology and End Results (SEER) registry. As a result, a cohort approach to assess the risk of angiosarcoma and other sarcoma following breast cancer is not feasible and a retrospective case-control study design was used instead.

Subjects were women who were 25 years of age or older when diagnosed with cancer in LAC during the period 1972–1995, and who were residents of LAC at the time of diagnosis. The study was designed to evaluate the risk of angiosarcoma associated with a history of invasive breast cancer; however, we also studied other subtypes of sarcomas for comparison. Four case groups were defined and compared to a control group consisting of non-sarcoma female cancer patients. The first case group consisted of 20 females diagnosed with angiosarcoma (lymphangiosarcoma and haemangiosarcoma; ICD-O 9120 and 9170) (Percy et al, 1991) of the upper extremity. The second consisted of 48 females diagnosed with lymphangiosarcoma or haemangiosarcoma of the chest and breast. In these two groups, other types of angiosarcoma, such as Kaposi's sarcoma, malignant haemangioendothelioma and malignant haemangiopericytoma, were excluded. The third and fourth case groups consisted of female patients with other soft tissue sarcomas, (excluding osteosarcomas; ICD-O 8800–8920) (Percy et al, 1991) located on the upper extremity ($n$ = 243) or the chest or breast ($n$ = 323) respectively. The control group ($n$ = 266 444), consisted of all female patients diagnosed at 25 years of age or older during the same time period with a primary malignancy other than sarcoma. We excluded patients with ovarian cancer, endometrial cancer and second primary breast cancer from the control group because of the known association of these cancers with a first primary breast cancer (Ederer et al, 1963; Harvey and Brinton, 1985). The case and control groups were then compared with respect to having had a prior primary invasive breast cancer diagnosed and reported to the registry. Since the registry collected details of the extent of disease and type of treatment only on patients diagnosed after 1987, such details could not be used in analysis.

Maximum likelihood estimates of the odds ratio (OR) were calculated using multivariate unconditional logistic regression methods; 95% confidence intervals (CI) were estimated using the standard error of the logarithm of the OR (Breslow and Day, 1980). Race (white vs non-white), age at diagnosis (25–39, 40–54, 55–64 and 65+) and year of diagnosis (1972–1979, 1980–1987 and 1988–1995) were included in the logistic regression models.

### RESULTS

The distribution of cases by histological type is shown in Table 1. Lymphangiosarcoma is the commonest type of angiosarcoma in the upper extremity (70%), while haemangiosarcoma makes up the majority of chest/breast angiosarcomas (98%). Among the other soft tissue sarcomas at these sites, fibrosarcoma, including malignant fibrous histiocytoma, is the most frequently diagnosed (72% in the upper extremity and 84% in the chest and breast).

We found that women diagnosed with breast cancer have a greatly increased risk of subsequent upper arm angiosarcoma, compared to women who have never been diagnosed with breast cancer (Table 2). After adjustment for age and year of sarcoma diagnosis, and race, the observed relative risk exceeds 59 (adjusted OR = 59.3, 95% CI = 21.9–152.8). The risk of subsequent angiosarcoma of the chest or breast itself is also strongly and significantly increased among women with a history of invasive breast cancer (adjusted OR = 11.6, 95% CI = 4.3–26.1), as is the risk of other non-vascular soft tissue sarcomas of the chest and breast (adjusted OR = 3.29, 95% CI = 1.7–5.8). However, non-vascular sarcoma of the upper extremity was not found to be associated with a previous breast cancer diagnosis.

Of the eight upper extremity angiosarcoma patients with a documented previous breast cancer, five tumours occurred on the same side as the breast cancer, one was reported to occur in the opposite upper extremity (see below), and the laterality of the original breast cancer was unknown for two patients. Among the six chest or breast angiosarcoma patients with a prior history of breast cancer, the laterality of the sequential tumours was the same in two, and unknown in the remaining four patients. The mean interval between the original breast cancer diagnosis and the subsequent angiosarcoma was 9.7 years for patients with upper extremity tumours and 4.4 years for those with chest or breast tumours respectively.

The prior breast cancers in patients with upper extremity angiosarcoma were diagnosed in an earlier time period compared to prior breast cancers in control patients (mean years of diagnosis 1976 vs 1981 respectively), even though the mean age at breast cancer diagnosis was similar in the two groups.

**Table 1** The distribution of female patients with angiosarcomas and other sarcomas, by histology, anatomic site and frequency of prior breast cancer diagnoses. Patients were diagnosed in Los Angeles County between 1972 and 1995; those diagnosed with sarcoma under 25 years of age were excluded

| Histological subtype | Upper extremity | | Chest or breast | | Other sites | |
|---|---|---|---|---|---|---|
| | No. (% total) | History of breast cancer | No. (% total) | History of breast cancer | No. (% total) | History of breast cancer |
| Angiosarcoma (ICD-O code) | | | | | | |
| Hemangiosarcoma 9120 | 6 (30%) | 4 | 47 (98%) | 6 | 62 (94%) | 0 |
| Lymphangiosarcoma 9170 | 14 (70%) | 4 | 1 (2%) | 0 | 4 (6%) | 0 |
| Total | 20 (100%) | 8 | 48 (100%) | 6 | 66 (100%) | 0 |
| Soft tissue sarcoma | | | | | | |
| Fibrosarcoma 8800–8841 | 174 (72%) | 1 | 270 (84%) | 9 | 974 (35%) | 2 |
| Liposarcoma 8850–8881 | 43 (18%) | 0 | 26 (8%) | 0 | 393 (15%) | 0 |
| Leiomyosarcoma 8890–8900 | 26 (10%) | 2 | 27 (8%) | 2 | 1405 (50%) | 0 |
| Total | 243 (100%) | 3 | 323 (100%) | 11 | 2772 (100%) | 2 |

© 1999 Cancer Research Campaign

**Table 2** Odds ratios (OR) and 95% confidence intervals (CI) for the risk of having a prior breast cancer among Los Angeles County female residents over 25 years of age diagnosed with angiosarcomas[a] and other sarcomas[b] of the upper extremity or chest between 1972 and 1995, compared to those with all other malignancies (controls)[c]

| | Exposure | | | | |
|---|---|---|---|---|---|
| | History of breast cancer 1972–1995 | No history of prior breast cancer | Unadjusted OR | Adjusted OR | 95% CI |
| Angiosarcoma[a] | | | | | |
| Upper extremity | 8 | 12 | 47.6 | 59.3 | 21.9–152.8 |
| Chest/breast | 6 | 42 | 10.2 | 11.6 | 4.3–26.1 |
| Other soft tissue sarcoma[b] | | | | | |
| Upper extremity | 3 | 240 | 0.9 | 1.1 | 0.3–2.8 |
| Chest/breast | 11 | 312 | 2.5 | 3.3 | 1.1–1.7 |
| All other malignancies[c] | 3634 | 262 810 | | | |

[a]Angiosarcoma (haemangiosarcoma and lymphangiosarcoma only). [b]Fibrosarcoma (including myxosarcoma and malignant fibrous histiocytoma), leiomyosarcoma, liposarcoma. [c]Excluding endometrial, ovarian and breast cancer, and all sarcoma except osteosarcoma. [d]Adjusted for age, race and year of diagnosis.

### DISCUSSION

Upper extremity angiosarcoma has long been reported to follow surgery for breast cancer (Stewart and Treves, 1948; Woodward et al, 1972; Maddox and Evans, 1981; Sanchez et al, 1989; d'Amore et al, 1990; Karpeh et al, 1991). The proportion of upper extremity angiosarcoma cases having had a prior breast cancer has ranged from 32% (Maddox and Evans, 1981) to 91% (Woodward et al, 1972). Follow-up studies (Ederer et al, 1963; Schottenfeld and Berg, 1971; Ewertz and Mouridsen, 1985; Harvey and Brinton, 1985; Taghian et al, 1991) of breast cancer patients have reported increased relative risks of any subsequent soft tissue sarcomas (including angiosarcoma) ranging from 1.8 (Taghian et al, 1991) to 4.5 (Schottenfeld and Berg, 1971), but there have been no attempts to quantify risk specifically for angiosarcoma, presumably because there were too few cases. We estimate this relative risk to be at least 59. The mean interval between breast cancer diagnosis and subsequent upper extremity angiosarcoma in our study was 9.7 years, similar in magnitude to that reported in other cases series (Woodward et al, 1972; Maddox and Evans, 1981; Janse et al, 1995).

In this case-control study, patients with cancer known to be associated with an increased risk of breast cancer were excluded from the control group in order to avoid biasing the result towards the null. While this may have caused the risk estimate to be slightly overestimated, we actually believe the relative risk of 59 to be a net underestimate due to the unavoidable under-ascertainment of breast cancers diagnosed prior to 1972. Upon review of the pathology reports of the 20 upper extremity angiosarcoma patients, we found evidence of breast cancer diagnosed prior to the existence of the registry for four of the 12 classified as 'unexposed'. One out of a sample of 34 'unexposed' non-sarcoma control cancer patients had evidence of a prior breast cancer, and none of the 42 'unexposed' patients with chest or breast angiosarcoma had such evidence. Thus, it is likely that the magnitude of the risk for upper extremity angiosarcoma is considerably higher than the observed 59-fold increase.

Post-mastectomy lymphoedema due to lymphatic blockage and/or radiation have been postulated as risk factors for the development of soft-tissue sarcomas following breast cancer (McClanahan and Hogg, 1954; Woodward et al, 1972; Maddox and Evans, 1981; Appelqvist et al, 1990; d'Amore et al, 1990; Karpeh et al, 1991). The majority of upper extremity lymphangiosarcoma cases had developed lymphoedema of the ipsilateral upper extremity following surgery for breast cancer and most, but not all, had received radiation therapy as part of their breast cancer treatment (Stewart and Treves, 1948; McClanahan and Hogg, 1954; Woodward et al, 1972; Maddox and Evans, 1981; Sanchez et al, 1989; Appelqvist et al, 1990; d'Amore et al, 1990; Rubin et al, 1990; Karpeh et al, 1991). The pathogenic mechanism by which lymphangiosarcoma develops is not known. However, lymphangiomatosis (proliferation of lymphatic vessels) is often seen in uninvolved areas of the affected oedematous extremity (Woodward et al, 1972), suggesting that lymphatic blockage stimulates the growth of lymphatic vessels, possibly via cytokines such as vascular endothelial growth factor (VEGF).

While one of these reported breast cancers (among the upper extremity angiosarcoma patients) had occurred contralateral to the subsequent angiosarcoma, this does not constitute evidence against a local mechanism. It is possible that this patient had bilateral breast cancer, with an initial primary occurring on the ipsilateral side prior to 1972 and therefore unidentified.

It is unclear if radiation contributes to the increase in risk. It could do so indirectly by causing axillary node sclerosis thereby producing lymphatic blockage and lymphoedema, or alternatively, by direct DNA damage. However, in the years of high-dose radiation, the upper extremity was protected from radiation by a lead shield. Since then, the trend has been to narrow and focus the beam of radiation on the tumour and axillary nodes, thereby reducing even further the likelihood of direct exposure of the upper extremity to ionizing radiation. A direct relationship between radiation therapy and subsequent sarcoma of the chest wall itself is more likely and has been suggested previously (Senyszyn et al, 1970; Kim et al, 1978; Ferguson et al, 1984; Souba et al, 1986; Taghian et al, 1991; Wiklund et al, 1991), although the majority of reported post-radiation sarcomas have been malignant fibrous histiocytoma, osteosarcoma and fibrosarcoma, rather than angiosarcoma. We could not address this question directly in the absence of detailed treatment data. However, the significant increase in both vascular and non-vascular chest wall sarcomas following a diagnosis of breast cancer certainly supports radiation exposure as a cause of sarcoma, although the magnitude of the risk

 © 1999 Cancer Research Campaign

**Table 3** Diagnosis date (year) of angiosarcoma patients with previous history of breast cancer

| | Diagnosis date of angiosarcoma | | Diagnosis date of prior breast cancer | |
|---|---|---|---|---|
| | Upper extremity angiosarcoma patients | Chest/breast angiosarcoma patients | Upper extremity angiosarcoma patients | Chest/breast angiosarcoma patients |
| 1972–1976 | 1 | 0 | 4 | 0 |
| 1977–1981 | 1 | 0 | 4 | 1 |
| 1982–1986 | 2 | 1 | 0 | 1 |
| 1987–1991 | 3 | 2 | 0 | 3 |
| 1992–1995 | 1 | 3 | 0 | 1 |

was higher for angiosarcomas (OR = 11.6) than for other sarcomas (OR = 3.3).

Although the relative odds for lymphangiosarcoma of the upper extremity following breast cancer is high, the absolute risk is low; the age-adjusted annual incidence in Los Angeles County females is only 0.02 per 100 000 women (USC-CSP, unpublished data). If lymphatic blockage and the resultant local lymphoedema were sufficient causes of upper extremity angiosarcoma, one would expect to have seen many more cases. An average of 6000 new breast cancers have been diagnosed annually in Los Angeles County between 1972 and 1995, and an estimated 13–37% of these patients are estimated to have developed long-term lymphoedema (Larson et al, 1986). Thus it is likely that additional factors are required. An inherited mutation in a tumour suppressor gene such as p53 (Malkin et al, 1990) might provide a permissive circumstance in which lymphatic blockage and the resultant lymphangiomatosis could lead to angiosarcoma. In fact, one patient with bilateral breast cancer and an upper extremity angiosarcoma has been tested in another context and found to have the BRCA1 mutation, 185delAG. Recent reports provide evidence that murine BRCA1 may have enzymatic activity involved with DNA repair (Somasundaram et al, 1997).

The eight upper extremity angiosarcoma patients who had a previous diagnosis of breast cancer reported to the registry had all been diagnosed with their breast cancer before 1981 (Table 3), and the four identified by pathology reports and not reported to the registry are presumed to have occurred before 1972. These breast cancers occurred during a period when the Halstead radical mastectomy with extensive axillary dissection was still in vogue. In contrast, the six patients who developed a subsequent angiosarcoma of the chest wall were diagnosed with breast cancer later, mostly in the late 1980s and early 1990s, during a period in which breast-conserving surgery with supplemental radiation therapy was more popular. These observations suggest that while the incidence of upper extremity angiosarcoma subsequent to breast cancer is probably decreasing, that of secondary angiosarcoma of the chest wall may be increasing. If the risk of developing angiosarcoma after radiation is associated with a constitutional characteristic, such as an inherited mutation, some of these highly fatal secondary cancers might be prevented by altering therapeutic strategies for those who are susceptible.

### ACKNOWLEDGEMENTS

This activity has been supported in part by the California Department of Health Services as part of its statewide cancer reporting program, mandated by Health and Safety Code Section 210 and 211.3. The ideas and opinions expressed herein are those of the authors, and no endorsement of the State of California, Department of Health Services or the California Public Health Foundation is intended or should be inferred. Contract number N01-CN-25403 of the Division of Cancer Prevention and Control, National Cancer Institute, National Institutes of Health, Department of Health and Human Services provided further support for this research.

### REFERENCES

Appelqvist P, Salmo M, Rissanen P and Wiklund T (1990) Response postmastectomy lymphangiosarcoma to radiotherapy: report of four cases. *Stahlenther Onkol* **166**: 194–198

Breslow NE and Day NE (1980) *Statistical Methods in Cancer Research*: *Vol I – The Design and Analysis of Cohort Studies*. IARC Scientific Publication No. 81, International Agency for Research on Cancer: Lyon d'Amore ESG, Wick MR, Geisinger KR and Frizzera G (1990) Primary malignant lymphoma arising in postmastectomy. *Am J Surg Pathol* **14**: 456–463

Ederer F, Cutler SJ, Goldenberg IS and Eisenberg H (1963) Causes of death among long-term survivors from breast cancer in Connecticut. *J Natl Cancer Inst* **30**: 933–947

Enzinger FM and Weis SW (1995) Vascular soft tissue tumors. In: *Soft Tissue Tumors*, Enzinger FM and Weis SW (eds), pp. 641–642. Mosby-Year Book: St Louis

Ewertz M and Mouridsen HT (1985) Second cancer following cancer of the female breast in Denmark, 1943–80. *Natl Cancer Inst Mongr* **68**: 325–329

Ferguson DJ, Sutton HG and Dawson PJ (1984) Late effects of adjuvant radiotherapy for breast cancer. *Cancer* **54**: 2319–2323

Harvey EB and Brinton L (1985) A second cancer following cancer of the breast in Connecticut, 1935–82. *Natl Cancer Inst Monogr* **68**: 99–112

Janse AJ, van Coevorden F, Peterse H, Keus RB and van Dongen JA (1995) Lymphedema-induced lymphangiosarcoma. *Eur J Surg Oncol* **21**: 155–158

Karpeh MS Jr, Caldwell C, Gaynor JJ, Hajdu SI and Brennan MF (1991) Vascular soft-tissue sarcomas: an analysis of tumor-related mortality. *Arch Surg* **126**: 474–1481

Kim JH, Chu FC, Woodard HQ, Melamed MR, Huvos A and Cantlin J (1978) Radiation-induced soft-tissue and bone sarcoma. *Ther Radiol* **4**: 501–508

Kuten A, Sapir D, Cohen Y, Haim N, Borovik R and Robinson E (1985) Post irradiation soft tissue sarcoma occurring in breast cancer patients: report of seven cases and results of combination chemotherapy. *J Surg Oncol* **28**: 168–171

Larson D, Weinstein M and Goldberg I (1986) Edema of the arm as a function of the extent of axillary surgery in patients with stage 1 and 2 carcinoma of the breast treated with primary radiotherapy. *Int J Rad Oncol Biol Phys* **12**: 877

Lowenstein S (1906) Der atiologische Zusammenhand zwischen akutem einmaligem Trauma and Sarkom. *Beitrage zur Klinsichen Chirurgie* **48**: 780–824

Mack TM (1977) Population-based cancer surveillance in Los Angeles County. *J Natl Cancer Inst* **47**: 99–101

Mack TM (1995) Sarcomas and other malignancies of soft tissue, retroperitoneum, peritoneum, pleura, heart, mediastinum and spleen. *Cancer* **75**: 211–245

© 1999 Cancer Research Campaign 

Maddox JC and Evans HL (1981) Angiosarcoma of skin and soft tissue: a study of forty-four cases. *Cancer* **48**: 1907–1921

Malkin D, Li FP, Strong LC, Fraumeni JF Jr, Nelson CE, Kim DH, Kassel J, Gryka MA, Bischoff FZ, Tainsky MA and Friend SH (1990) Germ line p53 mutations in a familial syndrome of breast cancer, sarcomas and other neoplasms. *Science* **250**: 1233–1238

Mark RJ, Poen J, Luu MT, Yao SF, Selch MT and Parker RG (1994) Postirradiation sarcomas: a single-institution study and review of the literature. *Cancer* **73**: 2653–2662

McClanahan BJ and Hogg L (1954) Angiosarcoma of the breast. *Cancer* **7**: 586–594

Percy C, Van Holten V and Muir C (eds) (1991) *International Classification of Diseases for Oncology*. World Health Organization: Geneva

Rubin E, Maddox WA and Mazur MT (1990) Cutaneous angiosarcoma of the breast 7 years after lumpectomy and radiation therapy. *Radiology* **174**: 258–260

Sanchez RB, Quinn SF, Walling A, Estrada J and White V (1989) Case report 569. *Skeletal Radiol* **18**: 485–487

Schottenfeld D and Berg J (1971) Incidence of multiple primary cancers. IV. Cancers of the female breast and genital organs. *J Natl Cancer Inst* **46**: 161–170

Senyszyn JJ, Johnston AD, Jacox HW and Chu FCH (1970) Radiation-induced sarcoma after treatment of breast cancer. *Cancer* **26**: 394–403

Somasundaram K, Zhang H, Zeng YX, Houvras Y, Peng Y, Zhang H, Wu GS, Licht JD, Weber BL and El-Deiry WS (1997) Arrest of the cell cycle by the tumour-suppressor BRCA1 requires the CDK-inhibitor p21WAF1/Cip1. *Nature* **389**: 187–190

Souba WW, McKenna RJ, Meis J, Benjamin R, Raymond AK and Mountain CF (1986) Radiation-induced sarcomas of the chest wall. *Cancer* **57**: 610–615

Steingaszner L, Enzinger FM and Taylor HB (1965) Hemangiosarcoma of the breast *Cancer* **18**: 352–361

Stewart FW and Treves N (1948) Lymphangiosarcoma in postmastectomy lymphedema: a report of six cases in elephantiasis chirurgica. *Cancer* **1**: 64–81

Taghian A, de Vathaire F, Terrier P, Le M, Auquier A, Mouriesse H, Grimaud E, Sarrazin D and Tubiana M (1991) Long-term risk of sarcoma following radiation treatment for breast cancer. *Int J Rad Oncol Biol Phys* **21**: 361–367

Wiklund TA, Blomqvist CP, Raty J, Elomaa I, Rissanen P and Miettinen M (1991) Postirradiation sarcoma: analysis of nationwide cancer registry material. *Cancer* **68**: 524–531

Woodward AH, Ivins JC and Soule EH (1972) Lymphangiosarcoma arising in chronic lymphedematous extremities. *Cancer* **30**: 562–572

*© 1999 Cancer Research Campaign*
